# Requirement of Plasminogen Binding to Its Cell-Surface Receptor α-Enolase for Efficient Regeneration of Normal and Dystrophic Skeletal Muscle

**DOI:** 10.1371/journal.pone.0050477

**Published:** 2012-12-11

**Authors:** Àngels Díaz-Ramos, Anna Roig-Borrellas, Ana García-Melero, Ana Llorens, Roser López-Alemany

**Affiliations:** IDIBELL – Institut d'Investigacions Biomèdiques de Bellvitge, Biological Clues of the Invasive and Metastatic Phenotype Research Group, L'Hospitalet de Llobregat, Barcelona, Spain; Goethe University, Germany

## Abstract

Adult regenerative myogenesis is central for restoring normal tissue structure and function after muscle damage. In muscle repair after injury, as in severe myopathies, damaged and necrotic fibers are removed by infiltrating inflammatory cells and then replaced by muscle stem cells or satellite cells, which will fuse to form new myofibers. Extracellular proteolysis mediated by uPA-generated plasmin plays a critical role in controlling inflammation and satellite-cell-dependent myogenesis. α-enolase has been described as plasminogen receptor in several cell types, where it acts concentrating plasmin proteolytic activity on the cell surface. In this study, we investigated whether α-enolase plasminogen receptor plays a regulatory role during the muscular repair process. Inhibitors of α-enolase/plasminogen binding: MAb11G1 (a monoclonal antibody against α-enolase) and ε-aminocaproic acid, EACA (a lysine analogue) inhibited the myogenic abilities of satellite cells-derived myoblasts. Furthermore, knockdown of α-enolase decreased myogenic fusion of myoblasts. Injured wild-type mice and dystrophic *mdx* mice were also treated with MAb11G1 and EACA. These treatments had negative impacts on muscle repair impairing satellite cell functions *in vitro* in agreement with blunted growth of new myofibers *in vivo*. Furthermore, both MAb11G1 and EACA treatments impaired adequate inflammatory cell infiltration and promoted extracellular matrix deposition *in vivo*, which resulted in persistent degeneration. These results demonstrate the novel requirement of α-enolase for restoring homeostasis of injured muscle tissue, by controlling the pericellular localization of plasmin activity.

## Introduction

Degeneration of skeletal muscle can result as a consequence of injury or disease, as in Duchenne Muscular Dystrophy (DMD), the most frequent neuromuscular disorder in boys, caused by a defect in the dystrophin gene [1]. Dystrophin is a large cytoskeletal protein that forms the dystrophin/glycoprotein complex at the sarcolemma, which links the cytoskeleton of myofibers to the extracellular matrix (ECM). The lack of dystrophin disturbs the assembly of the dystrophin/glycoprotein complex and causes instability of the muscle membrane, leading to muscle degeneration and myofiber loss. Although skeletal muscle has an extensive ability to regenerate, the permanent damage inflicted by absence of dystrophin leads to continuous cycles of degeneration, inflammation and regeneration [2].

Regeneration of adult skeletal muscle depends on muscle satellite cells, which are found beneath the basal lamina, in a mitotically quiescent state. In response to injury, satellite cells are activated, proliferate, and their progeny myoblasts differentiate into fusion-competent myocytes, which fuse with one another or with existing myofibers to restore normal tissue architecture [3]. The influx of inflammatory cells is also considered an essential event of the muscle regeneration process [4]. In addition, efficient muscle repair also requires the temporary deposition of ECM components (collagens, proteoglycans and laminin), in order to stabilize the tissue and serve as scaffold for new fibers. Formation and degradation of the ECM require the activity of several proteases, expressed during tissue repair [5]. If the regeneration process is compromised at any of its different stages, muscle tissue may be replaced by fibrotic tissue, associated with an impaired functional capacity.

The proteolytic conversion of the ubiquitous zymogen plasminogen (Plg) to the active plasmin (Pli) is an extensively used mechanism for the generation of extracellular proteolytic activity contributing to ECM degradation and tissue remodeling [6]. Plasminogen conversion into plasmin is exerted by two physiological plasminogen activators (PA): tissue-type (tPA) and urokinase-type (uPA). Plasmin is the major enzyme responsible for the dissolution of fibrin and most of the components of the ECM. Plasmin activity is tightly controlled at the level of PAs by plasminogen activator inhibitors (PAI-1 and PAI-2), and at the level of plasmin by α_2_-antiplasmin [6].

Work from numerous groups has clearly demonstrated that the localization of plasminogen and its activators on the cell surface, through association to specific cell membrane receptors, augments their catalytic efficiency [7], [8]. In this field, uPA is recruited to the cell membrane via a specific receptor (uPAR, CD87) [9]; several receptors for plasminogen have been described, including α-enolase [7], [10], annexin II [11] and histone H2B [12].

α-enolase is a glycolytic cytoplasmatic enzyme, considered a multifunctional protein and has been identified as a plasminogen receptor on the surfaces of several cell types [13]. On the cell surface, interaction of plasminogen with α-enolase enhances its activation by PAs, both forms plasminogen and plasmin bind to α-enolase and this binding protects plasmin from inhibition by α2-antiplasmin [7], [8].

Components of the PA system play important, yet distinct roles in muscle regeneration after injury, based upon the different muscle alterations observed in knock-out mice. Our group and others have shown that while both uPA and plasmin activities are necessary for skeletal muscle regeneration, tPA activity is dispensable [14], [15]. In contrast, PAI-1-deficient mice showed an improvement of muscle repair [16]. The important role of the PA system in muscular dystrophies has increased considerabily in the last years. For example, genetic loss of uPA exacerbated dystrophy and reduced muscle function in *mdx* mice [17]. Our group has previously demonstrated a role for plasmin in myogenesis *in vitro* as well as in skeletal muscle regeneration *in vivo* [15]. In vertebrates enolase exist as three different isoforms, α, β and γ, being β-enolase the majoritary form on the adult muscle [18]. β-enolase is expressed in proliferating adult myoblasts as well as in differentiated myotubes [19]. However, the α-enolase isoform has also been detected in muscle tissues and muscular cells [20], [21]. Furthermore, we have described that α-enolase is up-regulated in murine myoblasts C2C12 differentiation *in vitro*, and in muscle regeneration *in vivo* [22], raising thus the question of whether plasminogen receptors may also function in myogenesis and skeletal regeneration as a mechanism for regulating plasmin activity.

In order to examine the role of α-enolase in the pericellular generation of plasmin activity, we produced a monoclonal antibody, MAb11G1, that specifically blocked α-enolase/plasminogen binding and inhibited pericellular plasmin generation on peripheral blood neutrophils and monocytes [23]. In this study, we have investigated the role of α-enolase as a plasminogen receptor in muscle regeneration, using a combination of *in vivo* and *in vitro* models. We propose that abrogation of α-enolase/plasminogen interaction has a direct impact on inflammatory cell infiltration and satellite-cell-derived myoblasts differentiation.

## Materials and Methods

### Primary Cell Culture

Muscle Precursor Cells (MPCs) were obtained from muscles of young (4–8 weeks-old) normal mice as described [24]. MPCs were maintained on collagen-coated dishes in Ham's F10 medium supplemented with 20% fetal bovine serum (FBS) and 5 ng/ml bFGF (GM, growing medium). To induce differentiation, GM was replaced by differentiation medium (DM, DMEM supplemented with 2% horse serum) at myoblast subconfluence. All media were supplemented with 100 U/ml penicillin and 100 µg/ml streptomycin. Thioglycolate-induced mouse peritoneal macrophages were obtained as described [25].

### Inhibitors

Monoclonal antibodies MAb11G1 and MAb7H8 against α-enolase, produced in our laboratory [23]; ε-aminocaproic acid (EACA), Sigma; α2-antiplasmin, Loxo GmbH.

### Fusion assay

MPCs were cultured in 6-cm plates, 2.5×10^5^ cells/plate, in GM or DM. At the indicated time points, MPCs were fixed in 3.7% formaldehyde. Non-specific antibody binding was blocked with TNB buffer (NEN Life Science Products). Cells were then incubated with an antibody against Embryonic Myosin Heavy Chain (eMHC, F1652; Developmental Studies Hybridoma Bank) for 1 h at room temperature, and then incubated in biotinylated goat anti–mouse antibody (Jackson ImmunoResearch Laboratories). Number of nuclei in eMHC-positive cells was counted and expressed as a percentage of the total number of nuclei analyzed. The fusion index or myogenic index was determined by dividing the number of nuclei within myotubes (4 or more nuclei) by the total number of nuclei analyzed.

### Small interference RNA (siRNA)

siRNA was performed using Lipofectamine RNAiMAX (Invitrogen) according to the manufacturer's protocol. Briefly, 1×10^6^ cells were seeded in a 6-wells plate in GM and siRNA were used at 80 nM. The oligonucleotide sequences for the primer pairs used were: siRNA α-enolase (*ENO1*), 5′-UCA CAG GCU GUU GAG CAC AUC AAU A-3′ and 5′- UAU UGA UGU GCU CAA CAG CCU GUG A-3′; siRNA control, 5′-GUA AGA CAC GAC UUA UCG C-3′ and 5′- GCG AUA AGU CGU GUC UUA C-3′. After 24 hours, the medium was replaced by DM for further 48 hours.

### Quantitative Real Time PCR (qRT-PCR)

Total RNA was extracted using Ultraspect™ (BioTecx) isolation system, according to the manufacturer's instructions. RNA (1 µg) was reverse-transcribed to cDNA using Sensiscript enzyme (Invitrogen). Real-time PCR was used to measure specific mRNAs (Applied Biosystems 7300 Real-Time PCR system). Amplification mixtures (10 µl) contained the diluted cDNA sample, 5× LightCycler 480 Syber green Mastermix (Roche) and primers. Ribosomal protein L32 was used as endogenous control. Thermal cycling conditions included 10 min at 95°C before the onset of the PCR cycles, which consisted of 45 cycles at 95°C for 4 s, 62°C for 30 s and 72°C for 30 s followed by 1 cycle at 95°C for 10 min and 65°C for 1 min. The oligonucleotide sequences for the primer pairs used were: α-enolase (*ENO 1)*: 5′-AAC CCT GAA GTC ATC CTG CCT GTC-3′ and 5′- TTG CCA GAC CTG TAG AAC TCG GAG-3′; *myogenin*: 5′-GGT GTG TAA GAG GAA GTC TGT G-3′ and 5′-TAG GCG CTC AAT GTA CTG GAT-3′; *eMHC*: 5′ – AAA AGG CCA TCA CTG ACG C -3′ and 5′- CAG CTC TCT GAT CCG TGT CTC-3′ and ribosomal protein *L32*: 5′-AAC CCA GAG GAA TTG ACA AC-3′ and 5′-ATT GTG GAC CAG GAA CTT GC-3′. mRNA expression was calculated using the ΔC_t_ method [26].

### Mice and induction of muscle regeneration

Mice used were wild type (WT, 9- to 12-week-old) and *mdx* dystrophic mice (2- to 8-weeks-old), in C57Bl16 background (The Jackson Laboratory). All were maintained as a breeding colony and kept at room temperature with a natural night-day cycle. All animal experiments were approved by the Catalan Government Animal Care Committee (permit number 4520). Before manipulation, WT mice were anesthetized by an intraperitoneal injection of ketamin/xylacin. Muscular regeneration was induced by intramuscular injection of 150 µl of 10 µM cardiotoxin (CTX, Latoxan) in the gastrocnemius muscle group [17]. Once performed the injury, inhibitors were administered by subcutaneous injection every two days, in the injured region. Morphological and biochemical examinations of gastrocnemius muscles were performed at 4, 10 and 21 days post-injury (d.p.i.). The study in the *mdx* mice began when they were 2-weeks-old, before the onset of the disease. Inhibitors were administrated by intraperitoneal injection every two days. Mice were sacrificed at 30 and 60 days-old. Just before the sacrifice, blood was extracted by cardiac puncture. Diaphragm, tibialis anterioris and gastrocnemius muscles were analyzed. Six animals were used for each time point and inhibitor.

### Morphometric analysis

Cross-sections (10 µm) were collected from the mid-belly of muscles and stained with hematoxylin/eosin (H/E) (Sigma) and with Masson Trichrome (Sigma) using standard protocols. Images were acquired with an Olympus BX-60 using a Spot camera and Spot3.2.4 software (Diagnostic Instruments) and 10×0.25 NA, 20×0.40 NA, and 40×0.75 NA objectives (*Olympus*). The cross-sectional areas of entire muscles and individual myofibers were measured using the computer-assisted morphometric measurement ImageJ 1.35J (NIH) program. Muscle degeneration (%) was determined microscopically and expressed as a percentage of the total muscle area. Muscle fiber regeneration was determined microscopically and expressed as the percentage of total muscle fibers containing central nuclei present in the entire cross section of the muscle.

## Results

### α-enolase/plasminogen binding on muscle precursor cells is required for myogenesis

**Figure 1:**
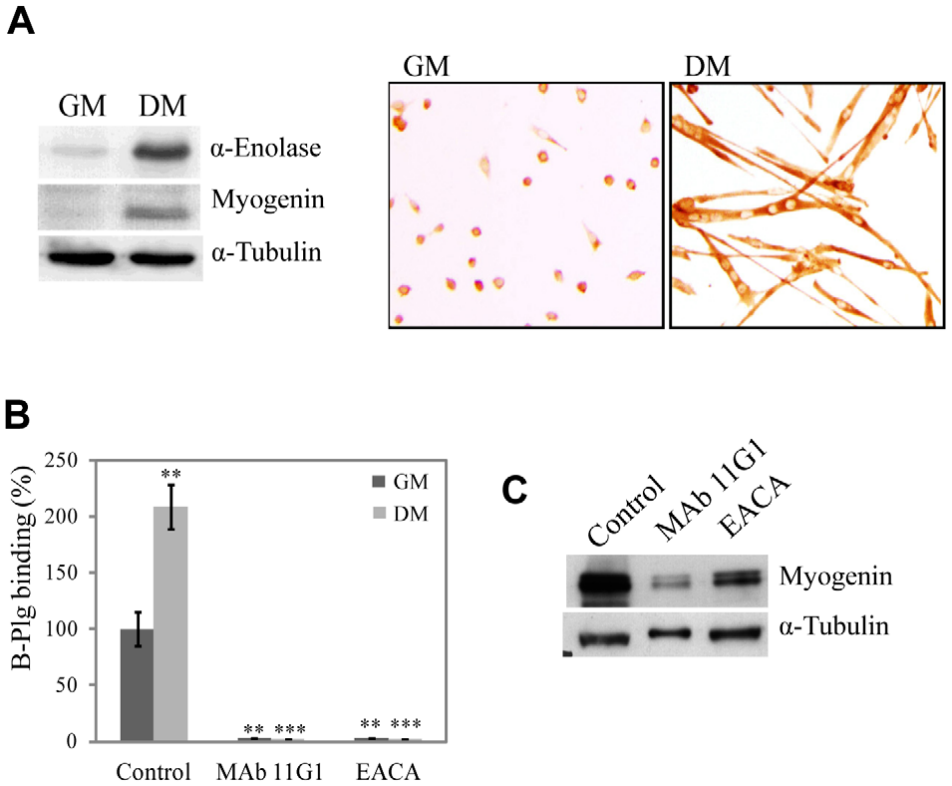
α-enolase expression and biotinylated-plasminogen binding are up-regulated in MPCs undergoing differentiation. **A**. α-enolase and myogenin expression on MPCs in Growing Medium (GM) and in Differentiation Medium (DM) for 48 hours. **B**. Byotinylated-plasminogen (B-Plg) binding to MPCs on GM and after 24 h on DM, in the presence of MAb11G1 and EACA. Results are expressed as a percentage of total biotinylated-plasminogen binding in control cells. Results are the mean +/− SEM (error bars) of three different experiments. ** *P*<0.005 *versus* B-Plg in GM, and *** *P*<0.005 *versus* B-Plg in DM. **C**. Expression of myogenin in differentiated MPCs in presence of MAb11G1 and EACA. A representative image of triplicates is shown.

We first addressed the question of the role of α-enolase on myogenesis by using primary cultures of muscle precursor cells (MPCs). α-enolase was expressed in proliferating MPCs cultured in growth medium (GM – with high serum content), and this expression was increased as myoblast differentiated and fused in differentiation medium (DM – with low serum content) (Fig. 1A). This increase was paralleled by a 2-fold induction of biotinylated-plasminogen binding to the cell surface after 48 h in DM (see Materials and Methods S1 and Fig. 1B). In the presence of 440 nM MAb11G1 (a monoclonal antibody against α-enolase, which selectively inhibits the α-enolase/plasminogen interaction) or 100 mM EACA (ε-aminocaproic acid, a lysine analogue that inhibits the capacity of plasminogen to bind to the cell surface through its Lysine Binding Sites), plasminogen binding to the cell surface was completely abolished (Fig. 1B), demonstrating the specificity of α-enolase in the plasminogen-binding to the MPCs surface.

**Figure 2:**
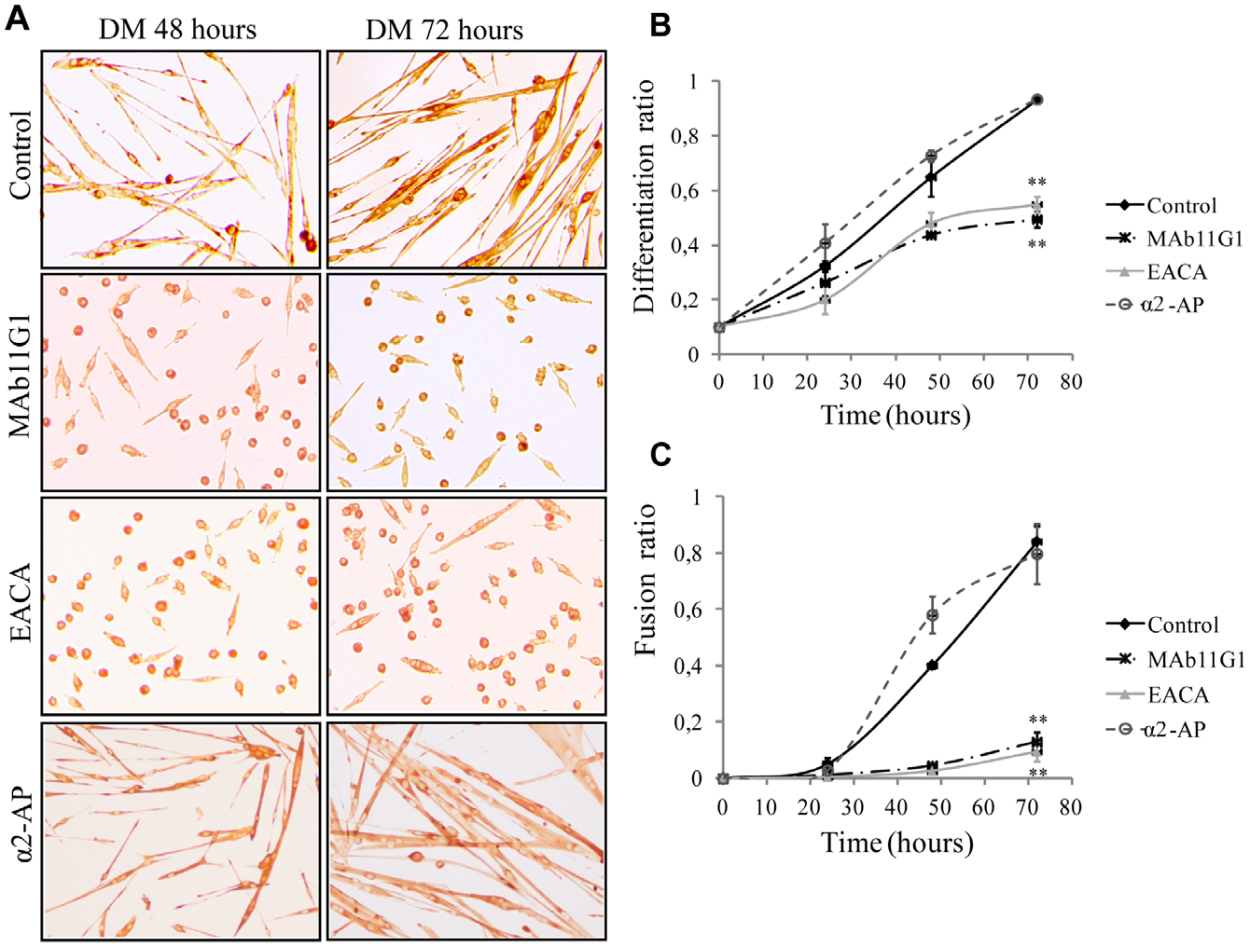
Inhibitors of α-enolase/plasminogen binding block myogenic differentiation and fusion. **A**. Immunocytochemical staining for eMHC of MPCs in DM in the presence of MAb11G1, EACA and α2-antiplasmin. **B**. Quantification of the Differentiation ratio (n° eMHC positive cells/total cells). **C**. Quantification of the Fusion ratio (n° eMHC positive cells fused in a myotube >4 nuclei/total cells) or Myogenic Index. Results are mean +/− SEM (error bars). ** *P*<0.005. Experiments were performed in triplicates.

We next used these inhibitors to assess whether the role of plasmin(ogen) in myogenesis is dependent on its capacity to associate to the cell surface. When MPCs were cultured under DM for 48 h, the addition of MAb11G1 or EACA, produced an important decrease in the expression of the differentiation-specific marker myogenin (Fig. 1C). Furthermore, an immunocytochemical assay (Fig. 2), using an antibody to embryonic Myosin Heavy Chain (eMHC) to stain differentiated myocytes/myotubes demonstrated that treatment with MAb11G1 and EACA inhibited the differentiation ratio by 46.9% and 41.05%, respectively, after 72 h in DM, compared to control cells (Fig. 2A and 2B). Myotube formation was also severely impaired by inhibition of α-enolase/plasminogen binding (Fig. 2A and 2C). At 72 h in DM, MAb11G1 and EACA inhibited the fusion index (polynucleated myotubes containing more of 4 nuclei), respectively by 82.59% and 86.97%. Furthermore, the addition of 100 nM α2-antiplasmin, the physiological inhibitor of plasmin, produced no inhibition of the differentiation or fusion ratio. The lack of inhibitory abilities of α2-antiplasmin is consistent through it is described that once plasmin is bound to its receptor, it remains protected against its inhibitors [7], [8]. These results suggest that plasmin activity needs to be pericellularly concentrated through α-enolase association for myogenic differentiation and fusion to occur efficiently; indeed, interference with this association severely compromised both myogenic processes.

**Figure 3:**
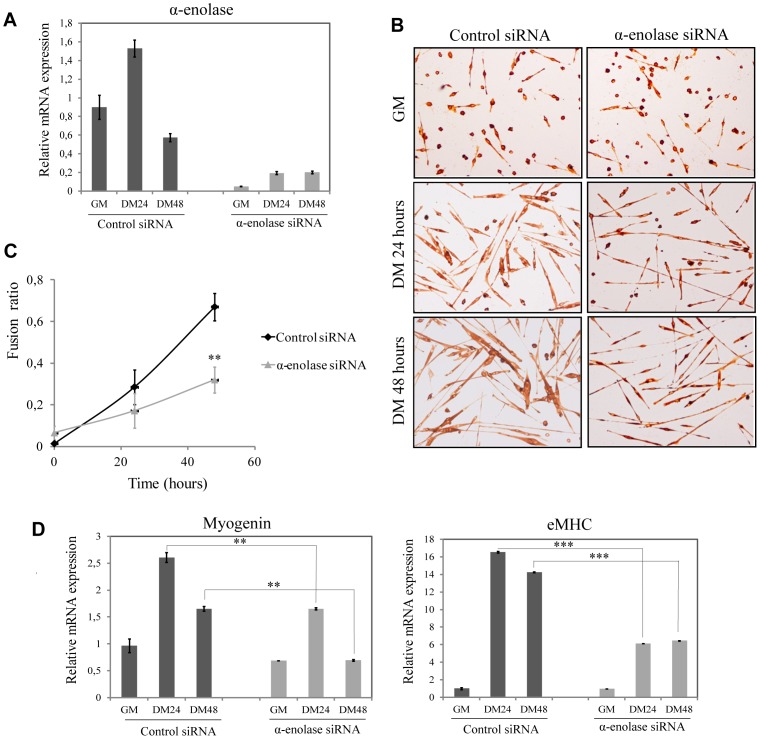
Knockdown of α-enolase by siRNA reduces MPCs differentiation. **A**. qRT-PCR to determine the relative mRNA quantity of α-enolase after siRNA transfection. **B**. Immunocytochemical staining for eMHC of MPCs transfected with α-enolase siRNA or control siRNA, on GM and DM at 24 and 48 hours. **C**. Quantification of the Fusion ratio (n° eMHC positive cells fused in a myotube >4 nuclei/total cells) or Myogenic Index. ** *P*<0.005. **D**. qRT-PCR to determine relative mRNA quantity of the genes for myogenin and eMHC. Ribosomal protein L32 mRNA levels were used for normalization. ** *P*<0.01, *** *P*<0.005. Results are the mean +/− SEM (error bars) of three different experiments.

Next, MPCs were depleted of α-enolase by siRNA. Twenty four hours after transfection of 80 nM α-enolase specific siRNA duplexes, cells were cultured in differentiation conditions for different times. α-enolase expression was markedly reduced, compared with control siRNA (Fig. 3A). Depletion of α-enolase induced a decrease of myogenic fusion, with a reduced number of polynucleated myotubes and formation of smaller myotubes (Fig. 3B). Immunocytochemical staining of eMHC showed an inhibition of the fusion index (polynucleated myotybes) of 55.8% in α-enolase silenced MPCs after 48 hours of differentiation (Fig. 3C). Furthermore, expression of myogenic markers as myogenin and eMHC were reduced by 40.3% and 39.9% respectively (Fig. 3D) in α-enolase depleted MPCs, indicating that the myogenic ability of these cells was significatively reduced. Therefore, α-enolase expression is necessary for myogenic fusion of MPCs.

**Figure 4:**
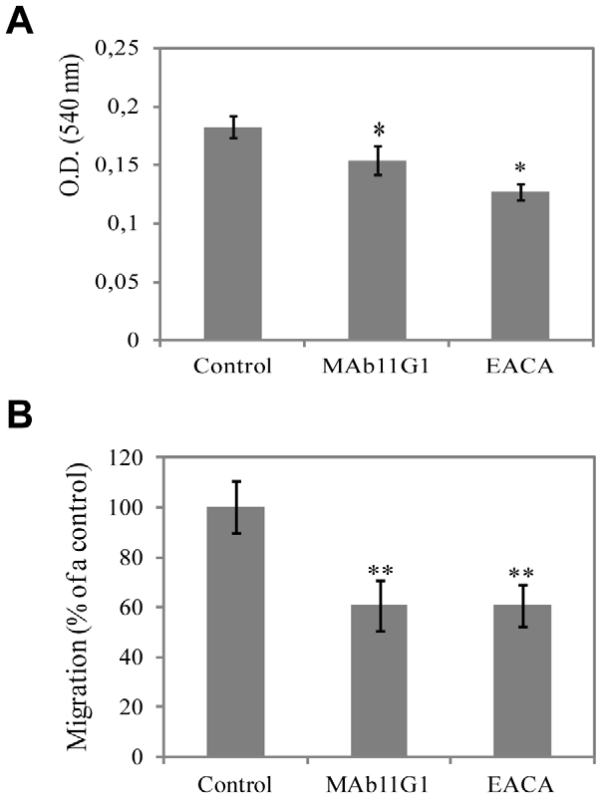
Inhibitors of α-enolase/plasminogen binding inhibits MPCs proliferation and migration. **A**. Proliferation of MPCs in the presence of MAb11G1 or EACA by the MTT proliferation assay. Results are mean +/− SEM (error bars) of quadriplicates. * *P*<0.01. **B**. MPCs migration in Transwells chambers, in the presence of MAb11G1 or EACA. Results are expressed as a percentage of control MPCs migration. Results are mean +/− SEM (error bars) of three different experiments. ** *P*<0.05.

Expansion of the myoblast population and further migration prior to their differentiation and fusion are critical steps in the regeneration of myofibers after injury. As shown in Fig. 4A, MAb11G1 induced a small but significative inhibiton on MPCs proliferation (15.6% inhibition), whilst the EACA inhibitory effect was more important (30.54%). Both MAb11G1 and EACA inhibited MPCs migration significatively (Fig. 4B). Altogether, these results indicate that α-enolase/plasminogen binding is critically required for satellite cell-derived myoblast proliferation, migration, differentiation and fusion.

### Selective interference of α-enolase/plasminogen association blunts muscle regeneration *in vivo*

**Figure 5:**
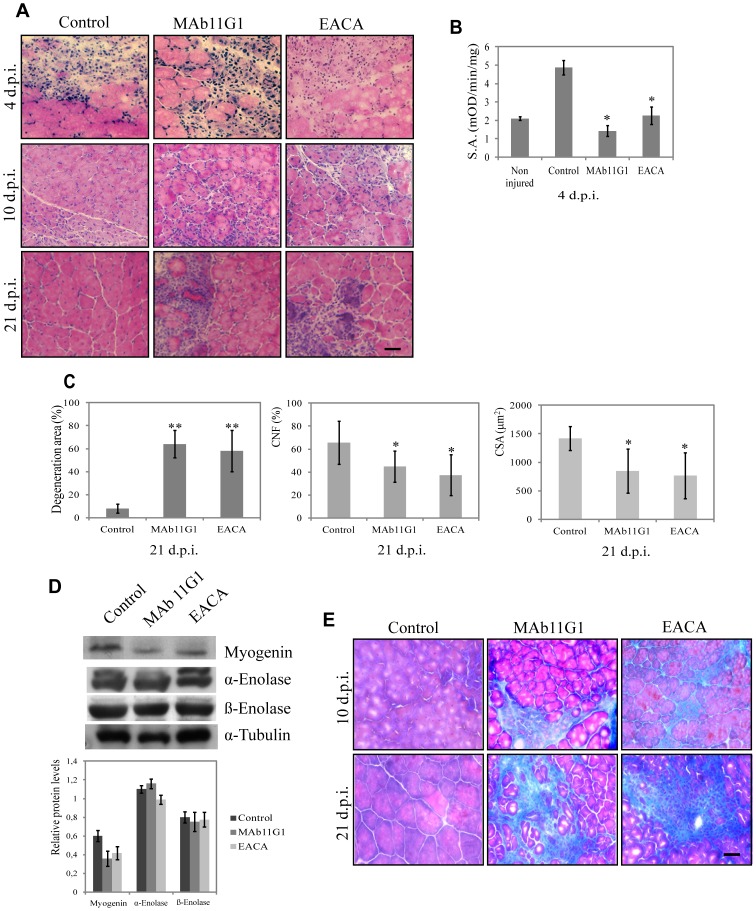
Inhibitors of α-enolase/plasminogen binding impede skeletal muscle regeneration after injury. **A**. Hematoxylin/eosin staining of cross-sections of gastrocnemius muscles of mice treated with inhibitors (MAb11G1, EACA or control) at different days post injury (d.p.i.) **B**. Plasmin specific activity (S.A.) of gastrocnemius muscle extracts, expressed as mOD/min/mg protein, as mean +/− SEM (error bars), * *P*<0.01. **C**. Percentage of degeneration area, percentage of central nucleated fibers (CNF) and mean fiber size of inhibitors-treated mice at 21 d.p.i., mean +/− SEM (error bars), ** *P*<0.005, * *P*<0.01. **D**. Western blotting of myogenin, α-enolase and β-enolase protein in tibialis anterioris muscle lysates. A representative image of triplicates is shown. Densitometric quantification of protein levels normalized with respect to α-tubulin is reported in the graphical diagrams. **E**. Trichrome staining for collagen deposits in gastrocnemius muscles, treated with the different inhibitors. Bars, 50 µm. *n* = 6 animals per group.

Knowing that the plasminogen-deficient mice showed a muscle regeneration defect [15], that α-enolase expression increases in regenerating muscle [22] and our demonstration that myoblasts require cell-surface-associated plasminogen for efficient myogenesis *in vitro*, we have investigated the role of α-enolase as a plasminogen receptor in muscle repair *in vivo*. The inhibitors described above were used in an *in vivo* model of injury-induced muscle regeneration by cardiotoxin (CTX) injection in the posterior limbs of 2-month-old mice. Inhibitors (440 nM MAb11G1, 100 mM EACA and physiologic serum used as control) were injected periodically (every 2 days) by subcutaneous injection (s.c.) in the injured hind-limbs, and regeneration analyzed at 4, 10 and 21 days post-injury (d.p.i.). Plasmin activity in muscle extracts at 4 d.p.i showed a 2.5-fold increase, compared to non-injured muscle, and was significatively decreased in inhibitors-treated mice (Fig. 5B). Histological analysis of injured muscles at 4 d.p.i. evidenced abundant myofiber necrosis and a highly disorganized tissue structure, irrespectively of the treatment (Fig. 5A). At 10 d.p.i. necrotic myofibers had been efficiently removed in control animals, while regeneration was well advanced, as indicated by the presence of small newly-formed myofibers with central nuclei (which identifies fibers undergoing regeneration). In contrast, muscles treated with MAb11G1 and EACA showed a highly disorganized architecture, with prominent edema, and fibrotic deposits within the enlarged intercellular space separating necrotic myofibers. At 21 d.p.i. control muscles showed well organized tissue architecture, characterized by large centrally-nucleated fibers. In contrast muscles treated with MAb11G1 and EACA still presented large necrotic areas, with high density of mononucleated cell infiltrates, and very small centrally-nuclei myofibers. In particular, at 21 d.p.i. treatment with MAb11G1 and EACA produced a 6.5-fold increase of the degeneration muscle area (Fig. 5C). A significant decrease of regeneration, as shown by the number of central nuclei fiber (CNF), and a decrease of the mean size of the myofibers (Fig. 5C) were observed. Treatment with MAb7H8, as isotype control, had no effect on the repair process (data not shown). Furthermore, a strong collagen accumulation in the muscle ECM, evidenced by trichrome staining, was found in muscles treated with MAb11G1 and EACA, at 10 and 21 d.p.i., while it was reduced in control mice at 10 d.p.i. and completely absent at 21 d.p.i. (Fig. 5E), suggesting an important role of the α-enolase/plasminogen binding in degradation of the transient ECM of injured muscle. Conversely, myogenin expression was reduced in extracts of mice treated with both inhibitors, when compared to control mice (Fig. 5D), whilst α-enolase expression was not affected. Thus, α-enolase/plasminogen interaction is required for the efficiency of the muscle regeneration process. β-enolase, the muscle-specific enolase isoform was also analyzed (Fig. 5D), and its expression remained unaltered in MAb11G1-treated mice and a slight decrease was observed in EACA-treated mice at 21 d.p.i.

**Figure 6:**
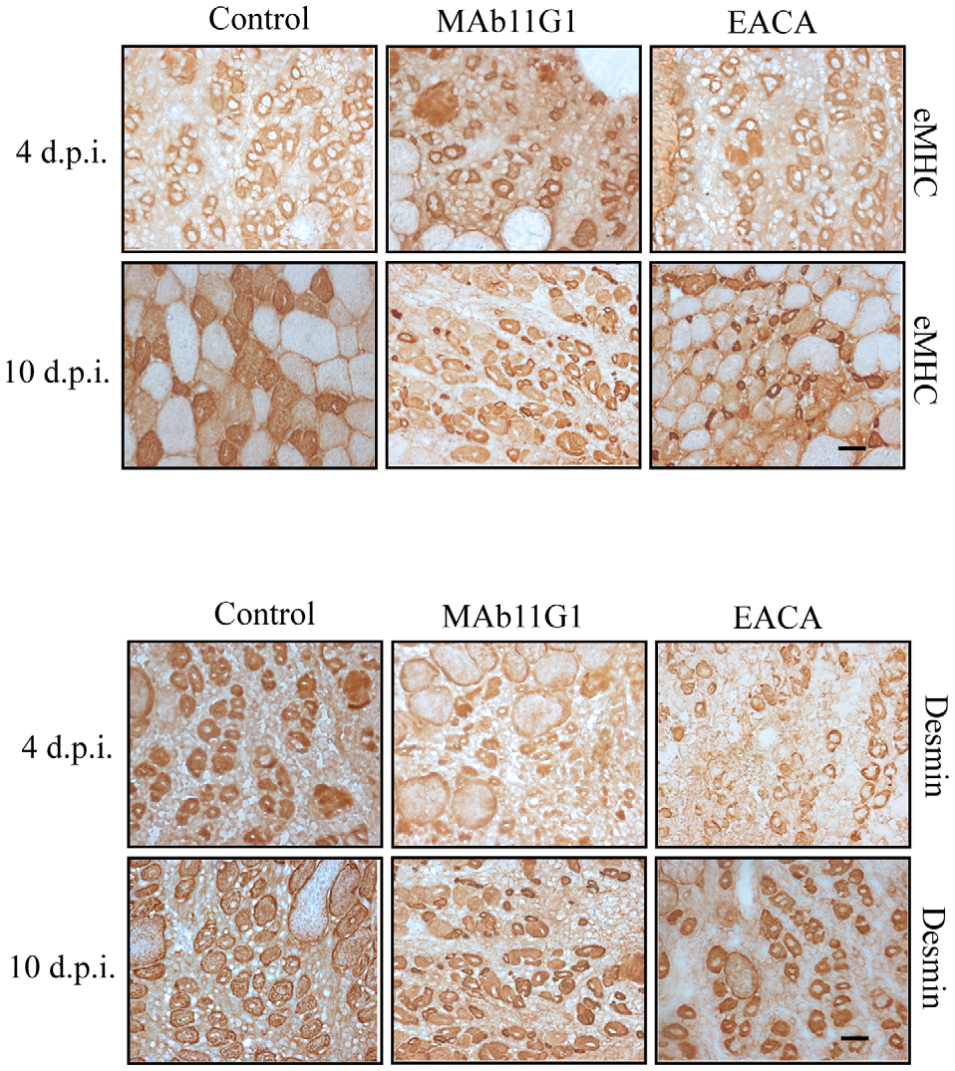
Inhibitors of α-enolase/plasminogen binding affect nascent myofibers (eMHC positive or desmin positive) into the damaged muscle. Serial cross-sections of gastrocnemious muscles at 4 and 10 days after cardiotoxin-induced injury were immunostained for eMHC and desmin. Representative images are shown. Bars, 50 µm. *n* = 3 animals per group.

Small but strongly expressing eMHC positive-fibers were present in MAb11G1- and EACA-treated mice at large stages after injury (10 d.p.i.), indicating a delayed myotube formation in mice treated with α-enolase/plasminogen binding inhibitors (Fig. 6). Furthermore, desmin (a marker for myoblasts and early stage myotubes) remained present in small fibers of MAb11G1- and EACA-treated mice, demonstrating the existence of more immature myofibers (Fig. 6). These results indicate that immature myofibers accumulate in the regenerating tissues, suggesting that α-enolase/plasminogen binding is necessary for the correct maturation of satellite cell-derived myoblasts.

### α-enolase/plasminogen binding is required for inflammatory cell infiltration in cardiotoxin-injured muscle

**Figure 7:**
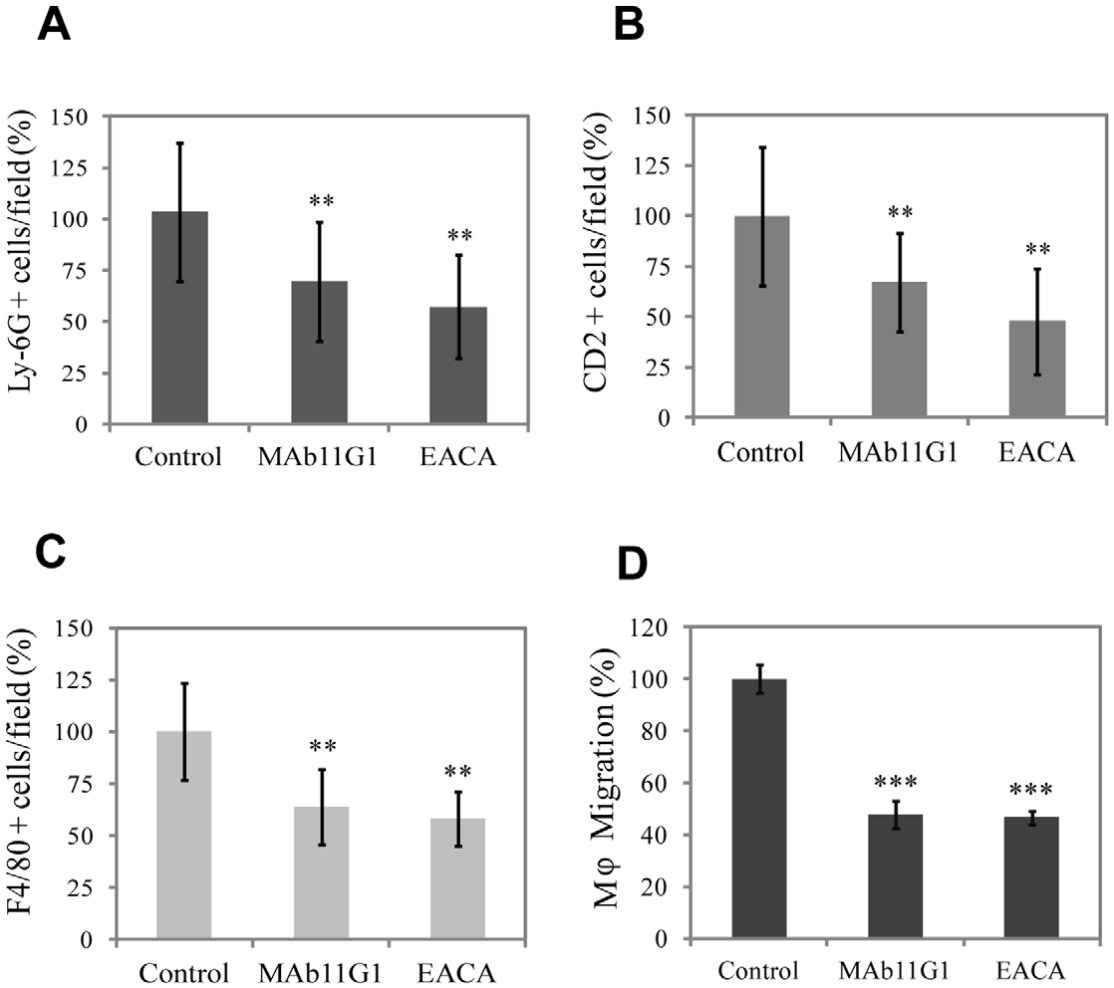
Inhibitors of α-enolase/plasminogen binding affect inflammatory cell recruitment to the damaged muscle. Immunohistochemical analysis of **A**, neutrophils (Ly-6G positive cells), **B**, lymphocytes (CD2 positive cells), and **C**, macrophages (F4/80 positive cells) in the cardiotoxin-injured gastrocnemius muscles at 4 and 10 days post injury. Results are expressed as a percentage of positive cells in the muscles of control- mice. *n* = 4 animals per group. **D**. Peritoneal macrophages migration in Transwells chambers, in the presence of inhibitors. Results are mean +/− SEM (error bars). ** *P*<0.01, *** *P*<0.005.

Although the impaired myogenic functions upon inhibition of the α-enolase/plasminogen axis could underlie the reduced growth of regenerating myofibers, they could not account for the persistence of necrotic infiltrates and degeneration in the treated muscles. Blood-borne monocyte-macrophages are recruited after injury to skeletal muscle during the inflammatory phase, that play a major role in the phagocytosis of tissue debris after muscel injury [27]. Furthermore, it has been previously shown that uPA- and plasminogen-deficient mice show an importatn decrease of the inflammatory response after muscle injury [14], [15]. Recently, α-enolase has been shown to promote plasminogen-mediated recruitment of monocytes to the acutely inflamed lung [28]; and contribute to peritoneal macrophage recruitment in response to thioglycolate [29]. Thus, we reasoned that a functional α-enolase/plasminogen axis might mediate the inflammatory response in damaged muscle. Accordingly, we analyzed the effects of MAb11G1 and EACA on the recruitment of neutrophils, T lymphocytes and macrophages to the dystrophic muscles, by immunofluorescency using specific antibodies for each type of cell. The number of neutrophils, lymphocytes and macrophages present in the injured muscles was reduced significativelly by MAb11G1 and EACA treatment (Fig. 7A, 7B and 7C). Consistent with these *in vivo* results, MAb11G1 and EACA also inhibited migration of freshly isolated primary macrophages in Transwell assays, indicating that macrophage migration depends on α-enolase/plasmin(ogen) binding and activity (Fig. 7D).

### Selective interference of α-enolase/plasminogen binding exacerbates muscular dystrophy in *mdx* mice

**Figure 8:**
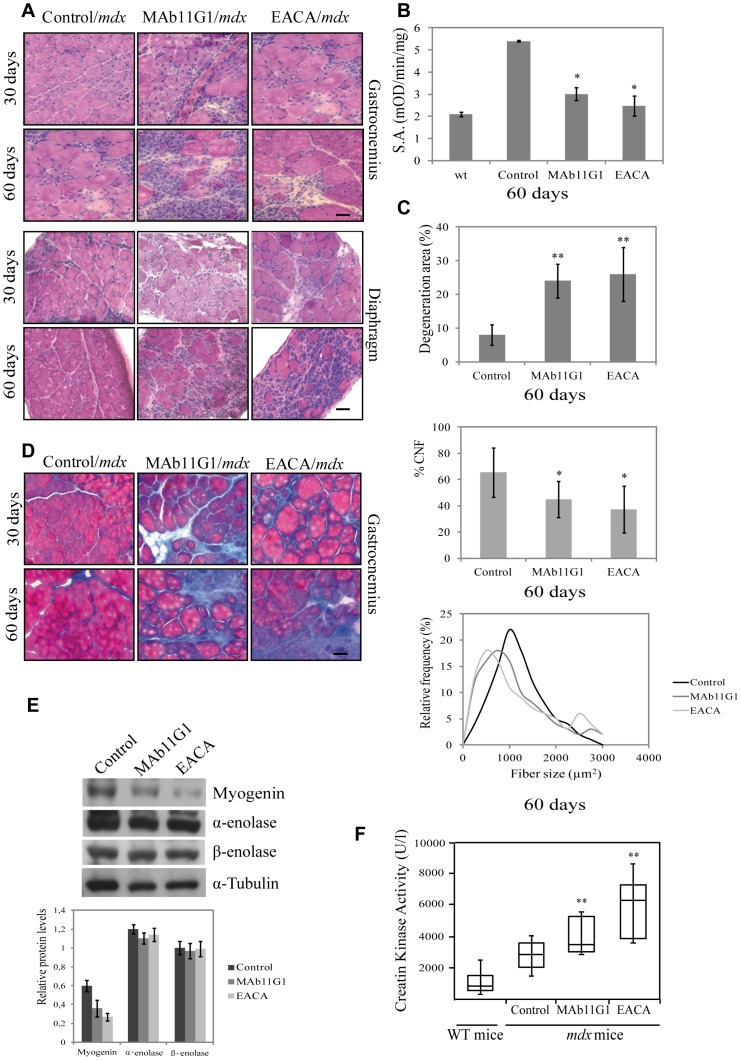
Inhibitors of α-enolase/plasminogen binding aggravate the myodystrophy of *mdx* mice. **A**. Hematoxylin/eosin staining of cross-sections of gastrocnemius muscles and diaphragms of *mdx* mice treated with the inhibitors (MAb11G1, EACA or control), until 30 and 60 days of age. **B**. Plasmin specific activity (S.A.) of gastrocnemius muscle extracts of treated-mice, represented as mean +/− SEM (error bars), * *P*<0.005. **C**. Percentage of degeneration area, percentage of central nuclei fibers (CNF) and frequency distribution of fiber size in gastrocnemius muscle of 60 day-old mice. ** *P*<0.005, * *P*<0.01. **D**. Trichrome staining of cross-sections of gastrocnemius muscles of *mdx* mice. **E**. Western blotting of myogenin, α-enolase and β-enolase protein in tibialis anterioris muscle lysates of mice treated or not with the indicated inhibitors. A representative image is shown. Densitometric quantification of protein levels normalized with respect to α-tubulin is reported in the graphical diagram. **F**. Creatine kinase levels in serum from 60 day-old mice. ** *P*<0.005. Bars, 50 µm. *n* = 6 animals per group.

It was important to analyze if the α-enolase/plasminogen axis might be functional in a muscular disease context, such as Duchenne muscular dystrophy (DMD), which courses with persistent tissue degeneration and fibrosis. We have previously shown an increase of α -enolase expression in muscle extracts of *mdx* mice (the most widely used animal model of DMD) when compared to WT mice [22]. Accordingly, we analyzed the effect of the inhibitors of α-enolase/plasminogen binding in the dystrophy progression in *mdx* mice. The inhibitors (800 nM MAb11G, 200 mM EACA), or physiologic serum, were injected periodically (every 2 days) intraperitoneally (i.p.). The treatment was initiated in 15 days-old *mdx* mice (before the onset of the disease), and was maintained until mice were 30 and 60 days old (during the peak of disease). At 30 days of age (treated with inhibitors for 15 days), obvious signs of myodystrophy were detectable in all treated mice, with disorganization of the muscle structure, presence of necrotic areas, inflammatory infiltrates and centrally-nucleated myofibers, indicating ongoing degeneration/regeneration processes (Fig. 8A). However, MAb11G1- and EACA-treated mice suffered a much more severe dystrophinopathy, characterized by an extensive myofiber degeneration and necrosis and increased presence of cellular infiltrates, compared to control mice. These differences were even more striking in 60 days-old mice (treated for 45 days). MAb11G1 and EACA treatment on mice of 60 days induced a 7-fold increase of the degenerating muscle area, approximately (Fig. 8C). Indeed, a decrease in regenerating fibers (CNF) and a decrease of fiber size were observed on MAb11G1 and EACA treated mice (Fig. 8C). The same effect of the inhibitors was even more evident in the diaphragm, considered the more affected muscle in the *mdx* mice (Fig. 8A). Compared to control mice, diaphragms of MAb11G1- and EACA-treated mice showed larger necrotic areas containing mononucleated cells and degenerating myofibers. Plasmin activity in *mdx* gastrocnemius muscles extracts showed a 2.8-fold increase, compared to WT muscles and was significativelly reduced in the inhibitors-treated mice (Fig. 8B).

The expression of the myogenic marker myogenin in MAb11G1- and EACA-treated mice was lower than in control mice, corroborating an impairment of the regeneration process (Fig. 8E). In contrast, no changes were observed in α-enolase expression, indicating that the observed effects are due to α-enolase function and not to α-enolase expression. No changes on β-enolase expression were observed in all treated mice (Fig. 8E). Furthermore, treatment with MAb11G1 and EACA produced an increase in collagen accumulation in dystrophic muscles, compared to control-treated mice (Fig. 8D).

Muscular creatine kinase (CK) expression is usually restricted to muscle. Upon sarcolemmal damage, CK muscular content is released to the blood stream, constituting a widely used biomarker of muscle damage [2]. Serum CK levels in the *mdx* mice were twice the levels in wild type mice and this value was duplicated and triplicated in MAb11G1- and EACA-treated mice, respectively, indicating that both inhibitors increased muscle damage in *mdx* mice (Fig. 8F).

**Figure 9:**
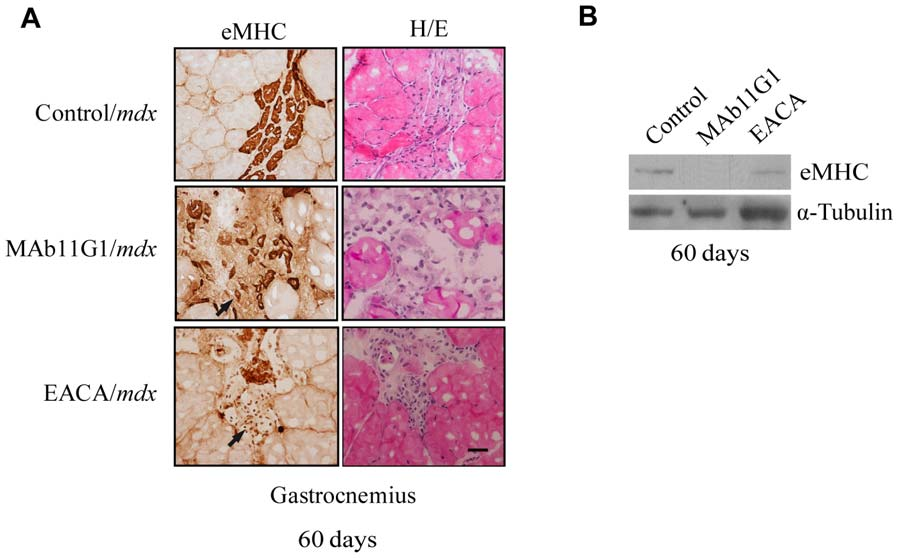
Inhibitors of α-enolase/plasminogen binding affect nascent myofibers (eMHC positive) to the damaged muscle. **A**. Serial cross-sections of gastrocnemious muscles of 60 days-old *mdx* mice were immunostained for eMHC or hematoxylin/eosin stained. Representative images are shown. Arrows indicate non-fused eMHC-positive cells. Bars, 50 µm. *n* = 4 animals per group. **B**. Western blotting of eMHC of gastrocnemius muscle lysates. A representative image of triplicates is shown.

Trying to define the identity of the mononucleated cells accumulated in the *mdx* degenerating muscles, an immunostaining of eMHC, a marker of myogenic differentiation, was performed. As shown in Figure 9A, strongly expressing eMHC fibers were observed in degeneration areas, charecterized by an accumulation of mononucleated cells, indicating that myogenic differentiation was taking place. MAb11G1- and EACA-treated mice showed an increase of mononucleated eMHC-positive cells, suggesting that the inhibitors treatment compromised the fusion process, in coincidence with the inhibition of myogenic fusion observed in muscle precursor cells (Fig. 2). Furthermore, eMHC expression was reduced in the inhibitors treated muscles, indicating that the myogenic fusion was compromised in these mice (Fig. 9B).

These results show that inhibition of α-enolase/plasminogen binding aggravates disease progression in dystrophic *mdx* mice.

### α-enolase/plasminogen binding is required for inflammatory cell infiltration in *mdx* dystrophic muscle

**Figure 10:**
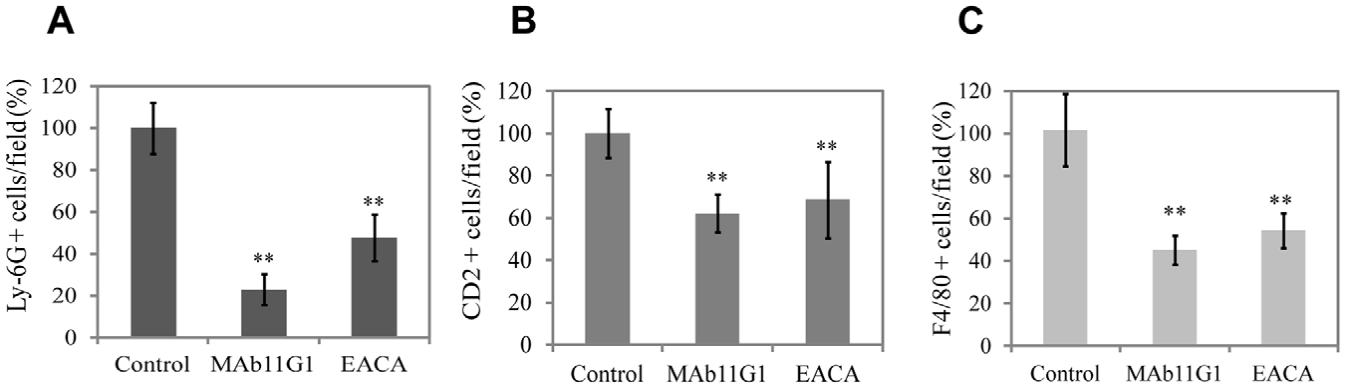
Inhibitors of α-enolase/plasminogen binding affect inflammatory cell recruitment to the damaged muscle. Immunohistochemical analysis of **A**, neutrophils (Ly-6G positive cells), **B**, lymphocytes (CD2 positive cells), and **C**, macrophages (F4/80 positive cells) in the gastrocnemius muscles of 60 days-old *mdx* mice. Results are expressed as a percentage of positive cells in the muscles of control- mice. *n* = 6 animals per group. ** *P*<0.005.

Muscle dystrophy is characterized by sustained levels of inflammatory cell infiltrates, particularly, neutrophils, macrophages and T cells [4], [17]. Recently uPA-mediated plasmin activity has been shown to be necessary to mount a proficient inflammatory response in *mdx* degenerating muscle [17]. Accordingly, we analyzed the effects of MAb11G1 and EACA on the recruitment of neutrophils, T lymphocytes and macrophages to the dystrophic muscles, by immunofluorescency using specific antibodies for each type of cell. The number of neutrophils, lymphocytes and macrophages present in dystrophic muscles was reduced significativelly by MAb11G1 and EACA treatment (Fig. 10). These results show that the recruitment of the major inflammatory cell types to dystrophic muscle was reduced by inhibition of α-enolase/plasminogen association.

## Discussion

Using genetically modified mice for uPA and plasminogen, we and others have shown that loss of uPA-mediated plasmin activity blunts muscle repair *in vivo* [14], [15], [17], [30]. However, whether plasmin activity requires cell-surface association for efficient muscle recovery, and in particular whether α-enolase functions as a cellular plasmin(ogen) receptor in this process, remained unknown. In this work, we demonstrate that α-enolase/plasminogen association regulates two timely-coupled processes in injured muscle: first, the recruitment of inflammatory cells for the resolution of tissue damage and, second, satellite-cell-dependent new myofiber formation.

Mice treated with inhibitors of α-enolase/plasminogen binding (MAb11G1 and EACA) showed an important defect in muscle regeneration, reminiscent of that observed in plasminogen-deficient mice and uPA-deficient mice [14], [15]. Furthermore, treatment with MAb11G1 and EACA aggravated myodystrophy in *mdx* mice, resembling the uPA-deficient *mdx* mice [17], which lacked plasmin activity, reinforcing the idea that inhibition of plasminogen binding to α-enolase impairs functional plasmin activity, necessary for maintaining muscle tissue homeostasis after damage.

Our results demonstrate that treatment with MAb11G1 and EACA affected several mechanisms in injured and dystrophic muscle. First, after a myotraumatism, an invasion of the damaged tissue by inflammatory cells is one of the first steps of the regeneration process, to degrade cellular debris and necrotic tissue, and to provide the initial cues for the subsequent proliferation, differentiation and fusion of satellite cells. Our results show a reduced recruitment of neutrophils, lymphocytes and macrophages to the dystrophic muscle of MAb11G1 and EACA-treated *mdx* mice. The same results were obtained in cardiotoxin-injured muscles. Thus, plasmin activity associated to the cell surface appears necessary for inflammatory cell recruitment to damaged muscle. Consistent with the impaired inflammatory response, we found an increased and persistent muscular degeneration in mice treated with α-enolase/plasminogen interaction inhibitors, probably due to the inability of the inflammatory cells, particularly macrophages, to phagocytose the necrotic tissue. Since uPA-mediated plasmin activity was recently shown to be necessary for a proficient inflammatory response in degenerating muscle [17], our results support the hypothesis that this proteolytic plasmin activity needs to be α-enolase associated to the surface of inflammatory cells to allow them to penetrate the damage muscle. Unresolved myofiber debris after MAb11G1 and EACA treatments was also accompanied by accumulation of intramuscular collagen deposits, which further contributed to the persistence of muscle degeneration when plasmin association to α-enolase was impaired.

Secondly, our results demonstrate that treatment with MAb11G1 and EACA directly affected injury-induced new myofiber formation *in vivo* as well as myogenesis *in vitro*. We found that satellite cell-derived myoblasts (i.e. MPCs) bound specifically plasminogen to the cell surface in an α-enolase dependent manner, and this binding increased during myogenic differentiation. This association was critical for myogenesis, since MAb11G1 and EACA inhibited satellite cell migration, proliferation, differentiation and fusion *in vitro*. In addition, down-regulation of α-enolase expression by siRNA reduced the size and number of myotubes and the expression of myogenic factors. Furthermore, an increase of differentiated (eMHC positive cells) but not fused cells were detected on MAb11G1 and EACA treated *mdx* muscles and injured muscles, suggesting that myogenic fusion was also impaired *in vivo*. These results demonstrate that plasmin activity is necessary for myogenesis, but, importantly, this activity needs to be cell-membrane associated. Our conclusions on the requirement for α-enolase-plasmin association for satellite cell-dependent myogenesis *in vitro* and *in vivo* are consistent with the impaired myogenic behaviour of myoblasts with selective interference with uPA and plasmin [14], [15], [31]. Expression of β-enolase was detected in both models of muscle regeneration analyzed, and it was unchanged upon the inhibitory treatment. The specificity of MAb11G1 on inhibing the plasminogen binding to α-enolase isoform and the decrease of myogenic fusion produced by knocking-down of α-enolase suggest that this is the isoform responsible of the effects observed on myogenesis.

Altogether, our findings show that treatment of *mdx* mice and cardiotoxin-injured muscles with inhibitors of α-enolase/plasminogen binding exacerbate muscle degeneration by both: inhibition of inflammatory cell recruitment and removal of necrotic tissue, and stimulation of collagen deposition. Thus it promotes the persistence of muscle degeneration, while inhibits muscle regeneration. In the *mdx* mice continous cycles of degeneration/regeneration and fibrosis development, coupled to attenuated satellite cell functions, are some of the important pathological causes of the progressive dysfunction and weakness in DMD patients [1]. Our results show that α-enolase/plasminogen interaction critically regulates these processes in the *mdx* mouse.

The work presented here shows that α-enolase is responsible of plasminogen/plasmin activity associated to the surface of critical regulatory cell types for muscular remodeling. Proteolysis associated to the cell surface is a usual mechanism in several physiological processes involving tissue remodeling. uPAR, the receptor for the urokinase-plasminogen receptor, concentrates urokinase-mediated plasmin generation on the cell surface, directing cell migration, adhesion and proliferation [9]. However, loss of uPAR *in vivo* neither affect the skeletal muscle degeneration/regeneration nor impair inflammatory recruitment; in addition, uPAR was not necessary for efficient myoblast differentiation and fusion *in vitro* [17], [32], indicating that uPAR is dispensable for efficient muscle repair. This reinforces the idea that α-enolase is the main functional receptor that concentrates proteolytic plasminogen activity on the cell surface during muscle tissue regeneration.

α-enolase has been described as plasmin(ogen) receptor in several cell types [7], [8], although the mechanism by which it is associated to the cell surface remains unknown, a characteristic shared with other described receptors for plasminogen as annexin II [11] and histone H2B [12]. Several post-translational modifications of α-enolase as phosphorylation, acetylation and methylation have been identified. For instance, an increase of acetylated lysines and methylated aspartic acid and glutamic acid residues of α-enolase were found in pancreatic ductal adenocarcinoma cells, compared to normal pancreatic duct cells [33]. Post-translational modifications of α-enolase have also been associated with autoimmunity to α-enolase in rheumatoid arthritis [34], [35]. However it remains to be determined how the phosphorylation, acetylation or methylation of α-enolase can affect its catalytic activity, localization on the cell surface or association with other proteins. Investigation of these modifications patterns in muscle regeneration and myodystrophies will provide insights into the role of α-enolase as plasminogen receptor in pathophysiological processes.

Other than its role in concentrating plasmin activity on the cell surface, the question of how α-enolase/plasminogen association affects muscle cells differentiation and migration remains still ongoing. The ability of plasminogen to induce intracellular signaling pathways activation has been described on several cell types [36]–[38]. DeSousa *et al.* have shown that plasmin(ogen) binding induce c-fos, egr-1 and α-enolase expression via the MEK/Erk pathway in fibroblasts and [39], [40]. We have also demonstrated that plasmin binding induce the activation of PI3Kinase and MEK/Erk pathways on muscle cells (Roig-Borrellas, unpublished results), through this issue remains to be further investigated.

Up-regulation of α-enolase has been described in several types of cancer, autoimmune diseases like rheumatoid arthritis and Alzheimer's disease [13], [41]. Recently, a proteomic meta-analysis of differently expressed 4.700 proteins, identified α-enolase as the first protein differentially expressed in mice and the second in human pathologies [42], suggesting that α-enolase could be considered as a marker of pathological stress in a high number of diseases. It is tempting to speculate than in many of these pathologies, α-enolase could exert one of its multiple functions, mainly as a plasminogen receptor, focalizing plasmin activity on the cell membrane and promoting ECM degradation/remodeling. The key role of plasminogen binding to α-enolase in myogenesis and muscle regeneration shown here constitutes a first exemple.

In summary, this study highlights the relevance of focalized pericellular proteolysis in tissue repair, and renders α-enolase/plasminogen association as a novel selective target for therapeutic interventions in muscle pathologies.

## Supporting Information

Materials and Methods S1 (DOC)
