# Supplementary material for: Requirement of Plasminogen Binding to Its Cell-Surface Receptor α-Enolase for Efficient Regeneration of Normal and Dystrophic Skeletal Muscle
Source: PLoS One. 2012 Dec 11;7(12):e50477. doi: 10.1371/journal.pone.0050477 (PMC3519827; doi:10.1371/journal.pone.0050477)
Supplement: Materials and Methods S1 — (DOC) [file pone.0050477.s001.doc]

# Supplemental Material and Methods

**Detection of biotinylated plasminogen in the cell surface**

For biotinylation, 1 µM plasminogen (Chromogenix) in bicarbonate buffer pH 8.0 was incubated during 1 min with 1mM N-succimidyl-ester (NHS)-biotin (Fluka) and dialyzed. Cells were incubated with biotinylated-plasminogen (100 nM), in absence or presence of the inhibitors, for 1 h at 37ºC. Cells were fixed with cold methanol, hydrated with phosphate buffer saline (PBS), incubated with Streptavidin-Horseradish Peroxydase and developed using ortophenylendiamine (OPD, Sigma).

**Proliferation assay.**

Cell proliferation was measured using the MTT (Thiazolyl Blue Tetrazolium blue, from Sigma) colorimetric assay. Briefly, 2x10^6^ MPCs/well were cultured in 96-wells plate, in GM for 24 hours, in the presence of MAb11G1 or EACA. Cells were then incubated with 5 mg/ml of MTT in culture medium for 3 h at 37ºC. The MTT formazan formed was diluted in DMSO and the absorbance at 540 nm was read. Each point was performed in quadruplicates.

**Migration assay**

Migration assays were performed on Transwells (8 µm pore size for MPCs and 3 µm pore size for macrophages, Becton Dickinson). Cells (5x10^4^) were added to the upper chamber of the Transwell in migration media (Optimem, Gibco). Insulin (170 nM, Sigma) and/or inhibitors were added to the lower chamber of the Transwell. After 24 h at 37ºC, cells on the filter’s upper surface were scraped off, while cells on the underside of the Transwell were fixed in 75% ethanol and stained with 2% Crystal Violet. The number of cells per high-power field that had migrated across the membrane was quantified (5 fields per filter). Results are expressed as the percentage of migrated cell compared to control migration in the absence of inhibitors.

**Muscle protein extracts and Western blot analysis**

Muscles were dissected out and pounded in an ice-cold Potter tube with 50 mM Tris-HCl pH 8.0; 150 mM NaCl; 1% NP-40; 5 mM EGTA and 1 mg/ml protease inhibitors cocktail (Roche). Protein concentration was determined in the supernatants using the Bio Rad protein assay. 50 µg of total protein were resolved by SDS-PAGE and transferred to Immobilon membranes. The antibodies used were: anti-α-enolase (H-300, Santa Cruz Biotechnology), anti-β-enolase (orb5157, Biorbyt), anti-myogenin (sc-576, Santa Cruz Biotechnology); anti-α-tubulin (DM1A, Sigma).

**Immunohistochemistry**

For the detection of inflammatory system cells, the following primary antibodies were used: for macrophages, anti-F4/80 (Serotec); for neutrophils, anti-Ly6G (BD Pharmigen), for lymphocytes, anti-CD2 (Caltag Laboratories). Immunohistochemistry was performed with the tyramide signal amplification cyanine 3 system (Perkin Elmer), following the described protocol [1]. Myofibers were stained using an anti-Embryonic Myosin Heavy Chain antibody (eMHC, F1652; Developmental Studies Hybridoma Bank) and a rabbit polyclonal antibody anti-desmin (clon D33, Dako); labeling was performed using the Vectastain ABC kit (Vector Laboratories), according to the maufacturer’s instructions. Control experiments without primary antibodies demonstrated that signals observed were specific (not shown).

**Plasmin activity of muscle extracts**

Plasmin content in muscle was measured using the S-2251 (Chromogenix)-based assay, done in triplicate in microtitration plates at 37ºC and calibrated with purified plasmin (Chromogenix). Muscle samples were lysed in a buffer containing 50 mM Tris-HCl pH 7.4, 1% Triton X-100 and 150 mM NaCl. 40 µg of muscle extracts were mixed with a buffer containing 0.1 M Tris-Hcl and 2 mM EDTA, pH 7.6 and 0.6 mM S-2251 as substrate. The generation of plasmin was detected by measuring the p-nitroaniline release from the substrate. Plasmin generation was detected as the increase in O.D. 405 nm, due to S-2251 hydrolysis.

**Serum Creatine Kinase activity**

Serum Creatine Kinase activity was measured with the indirect CK colorimetric assay kit and standards (Thermo Electron).

**Statistical analysis**

Quantitative data were analyzed by *t* test. *P* < 0.01 was considered statistically significant.

**References**

1. Suelves M, Vidal B, Serrano AL, Tjwa M, Roma J, et al. (2007) uPA deficiency exacerbates muscular dystrophy in MDX mice. J Cell Biol 178: 1039-1051.
